# Production of monoclonal antibodies reactive with ovine eosinophils

**DOI:** 10.1186/1471-2172-8-23

**Published:** 2007-09-27

**Authors:** Georgina M Sansome, Anna R Young, Els NT Meeusen, Robert J Bischof

**Affiliations:** 1 Zenyth Therapeutics Ltd, Biologicals Research Group, Richmond, Victoria, 3121 Australia; 2 Centre for Animal Biotechnology, School of Veterinary Science, The University of Melbourne, Victoria, 3010, Australia; 3 Animal Biotechnology Research Laboratories, Department of Physiology, School of Biomedical Sciences, Monash University, Clayton, 3800, Australia

## Abstract

**Background:**

There is strong evidence implicating eosinophils in host defence against parasites as well as allergic disease pathologies. However, a lack of reagents such as monoclonal antibodies (mAbs) specific for eosinophils has made it difficult to confirm the functional role of eosinophils in such disease conditions. Using an established mammary model of allergic inflammation in sheep, large numbers of inflammatory cells enriched for eosinophils were collected from parasite-stimulated mammary glands and used for the generation of mAbs against ovine eosinophils.

**Results:**

A panel of mAbs was raised against ovine eosinophils of which two were shown to be highly specific for eosinophils. The reactivity of mAbs 3.252 and 1.2 identified eosinophils from various cell and tissue preparations with no detectable reactivity on cells of myeloid or lymphoid lineage, tissue mast cells, dendritic cells, epithelial cells or other connective tissues. Two other mAbs generated in this study (mAbs 4.4 and 4.10) were found to have reactivity for both eosinophils and neutrophils.

**Conclusion:**

This study describes the production of new reagents to identify eosinophils (as well as granulocytes) in sheep that will be useful in studying the role of eosinophils in disease pathologies in parasite and allergy models.

## Background

Eosinophils have been proposed to play various roles in homeostasis, ranging from their involvement in tissue development to directing or facilitating innate and adaptive immune responses [1]. There is also a great body of evidence implicating eosinophils as central effector cells in parasitic and allergic disease. While there is sound evidence that demonstrates the contribution of eosinophils to host defence against parasitic infections [1,2], controversy still remains regarding the functional role(s) played by eosinophils in allergic diseases such as asthma [1]. This is largely based on inconsistencies between work in animal models and the human disease and the inability to effectively target eosinophils. For example, clinical resolution of human asthma could not be demonstrated by targeting eosinophils with anti-IL-5 therapy [3], despite the many studies in animal models that have shown the success of IL-5 neutralization in blocking experimental asthma [4-6]. It has subsequently been shown that pathways independent of IL-5 are relevant for eosinophil development or recruitment to sites of allergic inflammation [7].

The study of eosinophils in parasitic and allergic diseases has relied largely on the use of cytochemical stains that react with distinctive basic cytoplasmic granules of the eosinophil. Granule release or degranulation that commonly follows eosinophil recruitment into inflamed tissues, however, often limits use of cytochemical stains for the study of eosinophils.

In recent years, the use of monoclonal antibodies (mAbs) has been applied to the study of eosinophils and their functional role both *in vivo *and *ex vivo*. Eosinophils are known to express a range of membrane receptors enabling cell-cell communication, including receptors for adhesion molecules, immunoglobulins and soluble mediators such as cytokines and chemokines [1]. Some mAbs shown to be useful for identifying eosinophils, though not entirely specific for eosinophils, have included the IL-5 receptor, chemokine receptor-3 (CCR3) and the basic granule proteins, major basic protein (MBP) and eosinophil cationic protein (ECP) [1].

Sheep are the natural host to a range of helminthic parasites and therefore represent a relevant model to study the role of eosinophils in parasite immunity [8]. Sheep models of allergic inflammation have also been used for the study of eosinophils [9,10]. As in other species, ovine eosinophils have been shown to express a range of leukocyte surface markers including CD11a, CD11b, CD11c, CD18, CD29, CD44, CD45, CD49, and CD62L [9-11]. However, none of these proteins are expressed exclusively by eosinophils and to date, there are no mAbs that identify eosinophils in isolation in any species.

Given the close lineage relationship of granulocytes it is not surprising that a number of mAbs raised against cell surface components of eosinophils also show reactivity with other granulocytes. Most recently, Siglec-8 was reported to be the first identified eosinophil-exclusive surface receptor [12], however, a subsequent report demonstrated expression on basophils and mast cells [13]. Other examples include mAbs directed against eosinophil MBP and ECP that are also reactive with other granulocyte populations [14]. A major impediment to the generation of eosinophil-exclusive mAbs has been the inherent difficulty in gaining access to reasonable numbers of eosinophils of sufficient purity. The aim of this study was to use a sheep model of allergic inflammation to source large numbers of eosinophils and use these for the generation of mAbs specific for ovine eosinophils.

## Results

### Preparation of purified eosinophils

**Figure 1:**
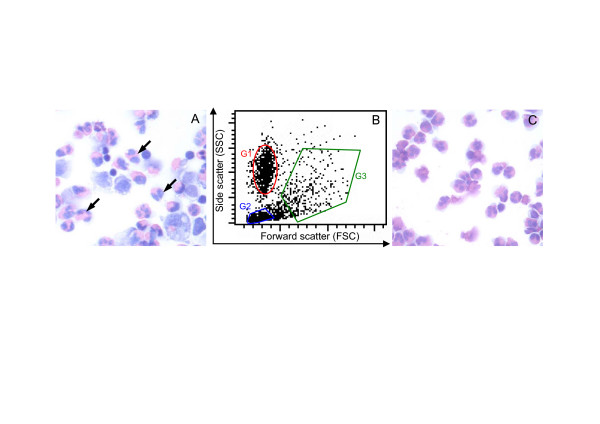
**Enrichment of eosinophils from mammary lavage leukocytes**. Mammary lavage leukocytes obtained 72 h post challenge with *Haemonchus contortus *L3 larvae. (A) Cytospin preparation of MAL cells stained with Wright's stain, showing mostly eosinophils (~80%; arrows) and lower number of lymphocytes (5–10%) and macrophages (10–15%). (B) The three populations of leukocytes in MAL were resolved on the basis of forward scatter *(FSC) *versus side scatter *(SSC)*.*G1*, eosinophils. *G2*, lymphocytes. *G3*, macrophages. (C) Cytospin preparation showing eosinophils which were gated and collected as in (B; *G1*) resulting in a highly enriched population of eosinophils (about 98%).

Eosinophils used for mAb production and screening were sourced from sheep mammary glands that were primed and stimulated with *Haemonchus contortus *L3 larvae. Differential cell counts of cytospots showed that mammary lavage (MAL) cells consisted of mostly eosinophils (80%), lymphocytes (10%) and macrophages (10%) (Figure 1A). MAL cells analysed by flow cytometry appeared as three distinct cell populations distinguished on the basis of their forward scatter (FSC) and side scatter (SSC) properties (Figure 1B), enabling mAbs to be screened for reactivity with the gated cell populations. The gated eosinophil population (Figure 1B; *G1*) was sorted using a flow cytometer and analysis of cytospins confirmed the high purity (>98%) of eosinophils (Figure 1C).

### Selection of monoclonal antibodies reactive with ovine eosinophils

MAbs reactive with sheep eosinophils were generated following mouse immunisations with preparations of eosinophil soluble lysate, granule extract, or whole live sorted cells.

Two fusions were performed from mice immunised with the soluble lysate and resulting hybridoma cell supernatants screened against eosinophil and neutrophil soluble lysates by Western blot. The mAb 1.2 (IgG_1 _istoype) was selected as it showed reactivity with eosinophils but not neutrophils.

Following fusions of spleen cells from mice immunised with eosinophil granule extracts, hybridoma supernatants were screened against eosinophil and neutrophil granule extracts by Western blot and eosinophil and neutrophil MAL cytospots by immunocytohemistry. Two clones reactive with eosinophils and neutrophils, designated mAb 4.4 (IgG_1_) and mAb 4.10 (IgG_1_), were characterised as granulocyte-specific markers.

A total of three fusions were performed with mouse spleen cells following immunisation of mice with whole eosinophils. Hybridoma supernatants were screened against MAL cells by flow cytometry and mAb 3.252 (IgG_1_) was chosen for its reactivity with eosinophils.

### Surface and intracellular staining of leukocytes with monoclonal antibodies against ovine eosinophils

Flow cytometry was used to assess whether the mAbs generated were reactive with cell surface or intracellular molecules (summary presented in Table 1). Furthermore, by gating on individual leukocyte populations within blood and MAL, the specificity of the mAbs was analysed. Reactivity was measured by the percentage positive reactivity relative to that observed with an isotype-matched control mAb, and the relative mean fluorescence intensity (RMFI).

**Table 1:** Reactivity of ovine granulocyte-specific monoclonal antibodies

		Antibody reactivity^§^			Cell reactivity	
		
mAb	Isotype	WB	Flow cytometry	IHC	eosinophil	neutrophil
1.2	IgG_1_	++^1^	++ (ic)	++ (cy,F)	++	-
3.252	IgG_1_	-	++ (su,ic)	++ (cy,F,P)	++	-
4.4	IgG_1_	++^2^	++ (ic)	++ (cy,F)	+	++
4.10	IgG_1_	++^2^	-	++ (cy,F)	+	++

^§ ^Western blot (WB) analysis (^1^reactivity detected on whole cell extracts; ^2^reactivity detected on eosinophil soluble and granule lysates); Flow cytometry analysis for surface (su) and intracellular (ic) reactivity; Immunohistochemistry (IHC) performed on cytospots (cy), and frozen (F) and paraffin-embedded (P) tissue sections fixed in 95% ethanol.

- no reactivity; + moderate reactivity; ++ strong reactivity

**Figure 2:**
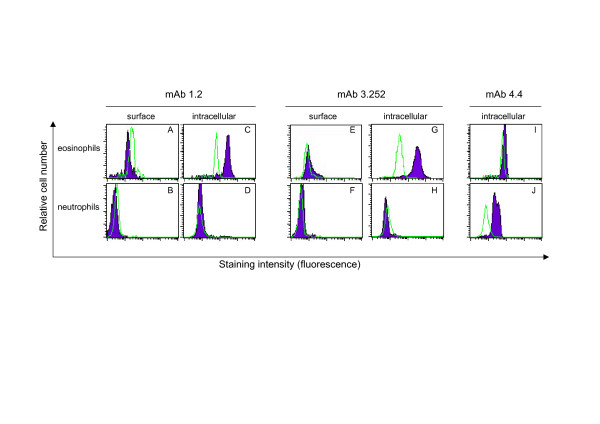
**Flow cytometrical analysis of mAb reactivity with eosinophils and neutrophils**. Flow cytometrical analysis of eosinophils and neutrophils stained for surface and intracellular expression with (A-D) mAb 1.2, (E-H) mAb 3.252 and (I, J) mAb 4.4. Positive staining is represented in each plot by a *solid histogram *while the reactivity with an isotype-matched control mAb is represented by an *open histogram*. Profiles shown are representative of three separate experiments.

MAb 1.2 showed no surface staining of any of the gated cell populations in peripheral blood or MAL including eosinophils (Figure 2A) and neutrophils (Figure 2B) when compared to an isotype matched control. However, following permeabilisation of cells to detect intracellular reactivity, mAb 1.2 showed clear positive intracellular staining of blood and MAL eosinophils (Figure 2C; >90%), but not neutrophils (Figure 2D), lymphocytes, monocytes or macrophages from peripheral blood, lymph node, MAL and bronchoalveolar lavage (BAL) samples (not shown).

In contrast, mAb 3.252 showed weak surface staining of MAL eosinophils (Figure 2E) and following permeabilisation greater than 98% of eosinophils showed intracellular staining (Figure 2G). Exclusive reactivity with eosinophils was confirmed by gating neutrophils (Figure 2F&H), lymphocytes, monocytes and macrophages from peripheral blood, lymph node, MAL and BAL samples which were unstained both on their surface and intracellularly (data not shown).

Expression of the antigen(s) identified by the mAbs 1.2 and 3.252 did not appear to be affected by eosinophil maturation and/or activation status as there was no change in staining intensity observed comparing eosinophils collected from bone marrow, peripheral blood and MAL (not shown).

The reactivity of the granulocyte mAbs (4.4 and 4.10) was examined on leukocytes in MAL and peripheral blood by flow cytometry. The mAb 4.4 showed no surface staining of cells, however, there was positive intracellular staining seen with both eosinophils (Figure 2I) and neutrophils (Figure 2J), with stronger relative staining apparent in neutrophils. In contrast, mAb 4.10 did not appear to show any reactivity with leukocytes by flow cytometry.

### Immunocytochemical staining and tissue distribution of eosinophils reactive with monoclonal antibodies 1.2 and 3.252

**Figure 3:**
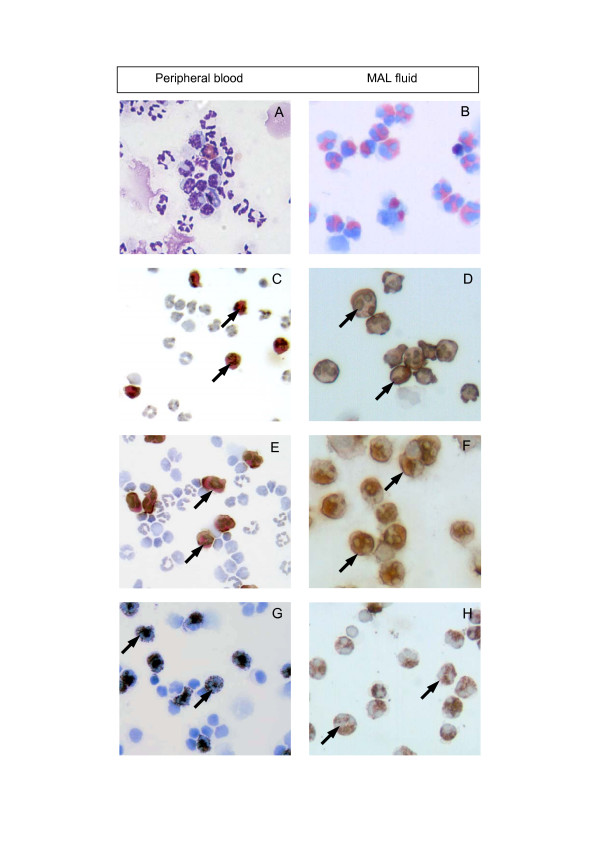
**Immunocytochemical staining of peripheral blood and mammary lavage cells with granulocyte-specific mAbs**. Cytospin preparations of peripheral blood and MAL leukocytes were stained with Wright's stain and mAbs 1.2, 3.252 and 4.10. (A, B) Cytospots stained with Wright's stain alone show the presence of eosinophils in peripheral blood and MAL. Positive staining of eosinophils (arrows) but not other leukocytes was seen with (C, D) mAb 1.2 and (E, F) mAb 3.252. MAb 4.10 showed intense staining (arrows) of eosinophils and neutrophils in (G) blood and (H) MAL cell preparations. Bound antibody was detected using the indirect immunoperoxidase technique, followed by weak H&E staining (Original magnification ×40).

The tissue distribution of eosinophils reactive with the mAbs 1.2 and 3.252 was assessed by immunocytochemistry on cytospin preparations and in tissues (summary presented in Table 1). Cytospin preparations of peripheral blood and MAL leukocytes confirmed the pattern of reactivity observed by flow cytometry (Figure 3). Peripheral blood and MAL eosinophils showed intense intracellular staining with the mAbs 1.2 (Figure 3C&D) and 3.252 (Figure 3E&F) while all other leukocytes were unstained. In contrast, the granulocyte-specific mAbs 4.4 and 4.10 showed intense staining of peripheral blood eosinophils and neutrophils in peripheral blood (Figure 3G) and MAL (Figure 3H).

**Figure 4:**
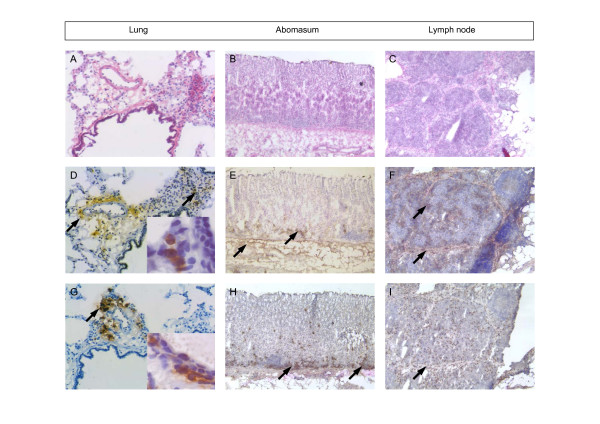
**Immunocytochemistry of ovine tissues stained with eosinophil-specific mAbs**. Tissue-infiltrating eosinophils were observed in frozen tissue sections following H&E staining in (A) lung tissue collected 48 h post-HDM allergen challenge, and (B) abomasum and (C) associated lymph node taken from a parasite-infected sheep. Immunoperoxidase staining of near serial tissue sections of lung, abomasum and lymph node with (D, E, F) mAb 1.2 and (G, H, I) mAb 3.252 shows staining associated with infiltrating eosinophils in each of the tissues examined (see arrows). Note the diffuse brown positive staining observed with mAb 1.2 compared with the more distinct reactivity of mAb 3.252. Higher magnification insets (in D and G) show detail of positively-stained eosinophils in allergen-challenged lung tissues.

Sheep tissues displaying high eosinophil levels due to parasite or allergic challenge (Figure 4) were sectioned and stained for reactivity with the mAbs 1.2 and 3.252. Lung tissue was collected following allergen challenge while abomasal (gut) and associated lymph node tissues were obtained from a gastrointestinal parasite infected sheep. Both mAbs showed optimal staining when tissues were fixed in ethanol, compared with paraformaldehyde or formalin. MAb 3.252 stained eosinophils equally well on frozen and paraffin embedded tissues, while staining with mAb 1.2 was optimal on frozen sections. Positive staining identified eosinophils in each of the tissues examined, with other leukocytes and tissue components unstained (Figure 4). However, staining with mAb 1.2 in many cases appeared more diffuse compared to reactivity seen with mAb 3.252. There did not appear to be any evidence of reactivity with tissue mast cells or basophils in normal or pathological tissues.

Cross reactivity of mAbs 1.2 and 3.252 with eosinophils from other species was assessed on cell and tissue preparations (normal and pathological) by immunocytochemistry. Both mAbs showed cross-reactivity with goat peripheral blood eosinophils while the mAb 3.252 also recognised cow eosinophils. No reactivity was detected with either mAb in cell and tissue samples sourced from murine (peripheral blood and various organs sourced from IL-5 transgenic mice) or human (peripheral blood and asthmatic airways) subjects.

### Western blot analysis with eosinophil-specific monoclonal antibodies

**Figure 5:**
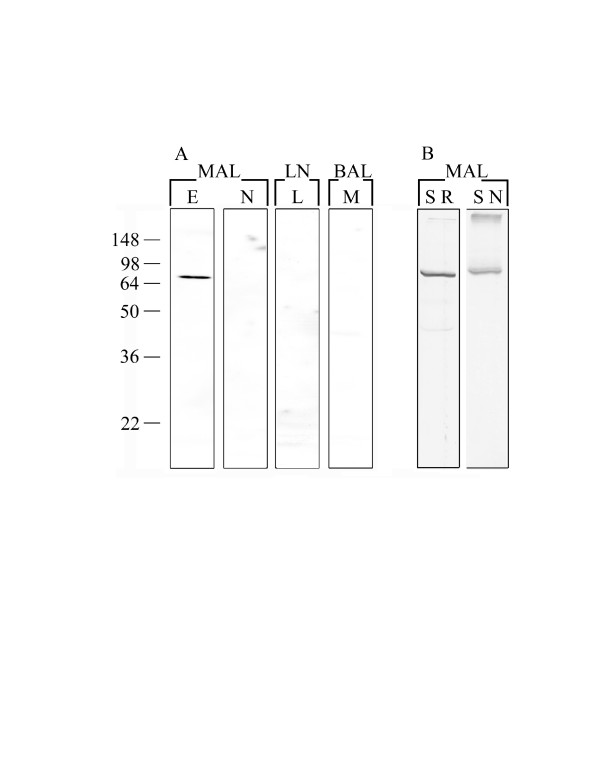
**Western Blot analysis of ovine cell preparations probed with the eosinophil-specific mAb 1.2**. (A) Equal numbers of cells (1 × 10^5^) enriched for MAL eosinophils (*E*), MAL neutrophils (*N*), lymph node (*LN*) lymphocytes (*L*) or BAL macrophages (*M*) were subjected to SDS-PAGE under reducing conditions followed by Western blot analysis using mAb 1.2. (B) Band detected by mAb 1.2 in a MAL eosinophil soluble lysate under reducing (*SR*) and non-reducing (*SN*) conditions.

The reactivity of the mAbs as assessed by Western blot analysis is summarised in Table 1. No reactivity of the mAb 3.252 was detected by Western blot. In contrast, Western blotting was used in the screening and selection of mAb 1.2 and could therefore be used to characterise the molecules recognised by mAb 1.2 in different sample preparations (Figure 5). Whole cell extracts from relatively pure (>80%) preparations of eosinophils, neutrophils, lymphocytes and macrophages were analysed by Western blot under reducing conditions. Extracts were prepared from an equal number of cells and identical blots probed with either mAb 1.2 or a negative control mAb. The eosinophil whole cell extract showed reactivity with a band of approximately 75 kDa, while no reactivity was detected in neutrophil, lymphocyte or macrophage extracts (Figure 5). Western blot analysis of the granulocyte-specific mAbs 4.4 and 4.10 showed reactivity with multiple bands (24–80 kDa) in eosinophil soluble and granule lysate preparations (not shown).

## Discussion

In the present study, we employed a sheep model of inflammation to provide access to large numbers of relatively pure eosinophils [9,15], required for mAb production and characterisation. Mice were immunised with either whole eosinophils, eosinophil soluble lysate or granule extract and the resulting mAbs were selected on the basis of their reactivity with eosinophils. These studies produced a panel of mAbs with reactivity for eosinophils alone (mAbs 1.2 and 3.252) and both eosinophils and neutrophils (mAbs 4.4 and 4.10).

The first eosinophil specific mAb 3.252 produced recognises eosinophils in bone marrow, peripheral blood and the inflamed mammary gland compartment, both by flow cytometry and immunocytochemistry. In fact, following their recruitment in response to allergic stimuli, all eosinophils (>98%) were stained by this mAb. Selection of mAb 3.252 was based on surface reactivity of mammary lavage (MAL) eosinophils, although the reactive protein was subsequently shown (by flow cytometry) to be present both on the cell surface and intracellularly. It is not uncommon for intracellular molecules to also be detected on the surface of cells, as has been shown to be the case with eosinophil derived neurotoxin (EDN), previously considered to be intracellular but recently identified at low levels on the surface of eosinophils [16]. The exclusive reactivity of mAb 3.252 at comparable levels on all eosinophils at different tissue sites suggests that this mAb would be suitable as a diagnostic tool for the discrimination of eosinophils in sheep.

The second eosinophil-specific mAb, mAb 1.2, proved useful for the discrimination of eosinophils by flow cytometry and immunocytochemical staining. There was little variation in the level of staining when comparing cells from bone marrow, blood and MAL, although in contrast to mAb 3.252, not all eosinophils stained positive with the mAb 1.2. A consistent, albeit small (5–10%), population of unstained cells was seen in all sites tested. Further, the 75 kDa protein identified in eosinophil lysates by mAb 1.2 could not be detected by Western blot in MAL fluid following eosinophil recruitment or *in vitro *in the culture supernatant of eosinophils stimulated to degranulate as measured by eosinophil peroxidase release (data not shown), suggesting the protein is not being released by eosinophils. This is in contrast to galectin-14, an exclusive product of eosinophils, which is released into the brochoalveolar (BAL) fluid after allergen challenge [17].

The application of mAb 3.252 and mAb 1.2 for immunocytochemistry allowed eosinophils to be detected in various tissues. Strong reactivity was detected in frozen tissue sections, while in fixed paraffin-embedded tissues, positive staining was detected after ethanol fixation but not with the use of formaldehyde-based fixatives. The widespread use of mAbs to identify eosinophils has included antibodies reactive with the IL-5 receptor, CC chemokine receptor-3 (CCR3) and the granule proteins MBP and ECP [1]. However, none of these markers are expressed exclusively by eosinophils. With the mAbs generated in the present study, and our earlier report describing a novel eosinophil-specific galectin [17], sheep are to date the only species in which eosinophil exclusive mAbs are available.

The identity of the antigens reactive with the eosinophil-specific mAbs generated in the present study is yet to be determined. The major constituents of the secondary granules, generally low molecular weight proteins, are amongst the best-characterised intracellular proteins of the eosinophil. These include the cytotoxic cationic granule proteins, as well as an array of pro-inflammatory cytokines, chemokines and lipid mediators [1]. It was found that mAb 1.2 identified a protein band at 75 kDa in eosinophil lysates, much greater than the molecular weight of known granule components. It seems that both mAb 1.2 and 3.252 do not react with specific granules but as yet unidentified eosinophil constituents. The identification of these antigens, a current focus in our laboratory, will be required to characterise the role of these molecules in eosinophil-associated pathologies.

The mAbs generated in the present studies will assist the study of ruminant eosinophils *ex vivo *and *in vivo*. With the use of appropriate models of parasite and allergic disease, these mAbs may be shown to be effective for the inhibition of eosinophils or their effector functions, and contribute to our understanding of the role of eosinophils in protection against infection and disease pathology.

## Conclusion

In this study, two mAbs that identify sheep eosinophil and neutrophil granules and two eosinophil exclusive mAbs have been produced. The mAbs 1.2 and 3.252 react with eosinophils but not lymphocytes, monocytes, neutrophils or macrophages. An eosinophil exclusive mAb has great potential as a tool for eosinophil research as to date there are limited reagents available for identifying eosinophils. Other mAbs generated here were found to be reactive for both eosinophils and neutrophils (mAbs 4.4 and 4.10). The study of immunological responses of sheep to parasite infections, where eosinophils are implicated, and in sheep models of allergic inflammation will now be greatly facilitated by the development of these eosinophil specific reagents.

## Methods

### Animals

Mature non-lactating merino ewes (2 years of age) were purchased from a commercial farm and treated with the anthelminthic Nilverm™ (Cooper's Animal Health, New South Wales, Australia) to eliminate any existing parasite infections. Sheep were housed in pens at the School of Veterinary Science, The University of Melbourne and fed dry pellets (Barastoc, Victoria, Australia) and water *ad libitum*. All experimental procedures and the collection of tissues and cells were approved by the Animal Experimentation Ethics Committee of The University of Melbourne, following guidelines set by the National Health & Medical Research Council (NH&MRC) of Australia.

### Mammary gland infusions and the collection of mammary lavage cells

For the collection of mammary lavage (MAL) leukocytes, mammary gland infusions were performed as previously described [9]. Briefly, for eosinophil recruitment into the gland, non-lactating Merino ewes were primed with weekly infusions of 5000 exsheathed *Haemonchus contortus (Hc) L3 *larvae, rested for 3–4 weeks followed by challenge of the gland with *Hc *larvae. Cells were retrieved 3 days post challenge with the infusion of sterile pyrogen free saline (PFS, Baxter Healthcare, New South Wales, Australia) and milking of the gland. For collection of neutrophils, mammary glands were infused with lipopolysaccharide (LPS) and cells collected 24 h later [15]. Cells were pelleted by centrifugation and washed in PFS. Cytospots were prepared and stained with Wright's stain (Sigma, New South Wales, Australia) to determine the proportion of eosinophils or neutrophils in the suspension.

### Preparation of cell suspensions and tissue samples

Peripheral blood leukocytes (PBL) were collected from the jugular vein of sheep and placed into a tube containing 80 mM ethylamine tetra-acetic acid-Na_2 _(EDTA). Cells were treated with Tris-buffered ammonium chloride (TAC; 0.17 M Tris/0.16 M NH_4_Cl pH 7.2) to lyse red blood cells. Lymphocytes were prepared from resected lymph nodes by gentle teasing with forceps in cold Dulbecco's Modified Eagles Medium (DMEM; Invitrogen, Life Technologies). Alveolar macrophages were obtained from bronchoalveolar lavage (BAL) fluid as described previously [10,18]. Eosinophils and neutrophils were prepared from MAL washings as described earlier.

In each case cells were washed three times in phosphate-buffered saline (PBS) before being resuspended at 1 × 10^7 ^cells/ml in SDS-PAGE reducing buffer and stored frozen prior to Western blot analysis, or in 2% bovine serum albumin (BSA, Fraction V; Trace Biosciences, Victoria, Australia)/PBS for flow cytometry analysis. Purity of the cell preparations was found to be greater than 80% in each case, as assessed on cytospots stained with Wright's stain.

IL-5 transgenic mice, displaying high levels of peripheral blood and tissue eosinophilia [19], were kindly provided by Dr. Lindsay Dent (Department of Microbiology and Immunology, University of Adelaide). Caprine and bovine peripheral blood for immunocytochemistry was provided by Dr Stuart Barber (Department of Veterinary Science, The University of Melbourne). Paraffin-embedded human lung and large airway tissue from an asthmatic patient were kindly donated by Dr. Alastair Stewart (Department of Pharmacology, The University of Melbourne).

### Preparation of soluble and granule extracts

MAL cells derived from *Hc *(eosinophils) or LPS (neutrophils) stimulated glands were washed three times in PBS before being lysed by repeated freeze thaw cycles of 1 × 10^7 ^pelleted cells. For the preparation of soluble lysates, lysed cells were resuspended in PBS and centrifuged at 100,000 *g *for 20 min. Granule extracts were prepared based on previously described methods [20]. Briefly, lysed cells were centrifuged at 13,000 *g *for 20 min, and pelleted granules exposed to 0.01 M HCl (pH 2) followed by sonication and final centrifugation (40,000 *g *for 20 min) to remove the released granule proteins from the insoluble material. Protein concentration of the resulting supernatants was determined using the bicinchoninic acid (BCA) assay (Pierce, Illinois, USA) and aliquots consisting of 10 μg and 50 μg of protein were stored at -20°C prior to mice immunisations. Identical extracts were separated by SDS-PAGE for hybridoma supernatant screening by Western blot analysis.

### Production of monoclonal antibodies

For the production of monoclonal antibodies (mAbs), 3 groups of BALB/c mice were immunised intraperitoneally (i.p.) with eosinophil preparations. Group 1 mice received 10 μg of eosinophil soluble extract in complete Freund's complete adjuvant (CFA; Sigma). Group 2 mice received 10 μg of eosinophil granule extract in CFA. The third group of mice (group 3) received an eosinophil rich preparation consisting of 2 × 10^7 ^MAL cells (~80–98% pure eosinophils) in 500 μl PBS. Subsequent injections were given at monthly intervals. Six weeks following the third immunisation, mice received 50 μg of protein (soluble lysate/group 1; granule extract/group 2) in PBS or a 100% pure (sorted by flow cytometry) preparation of eosinophils (group 3) intravenously (i.v.). Spleens were harvested 4 days following the final i.v. immunisation and fused with NS-1 myeloma cells using 50% polyethylene glycol 4000 (Merck, Darmstadt, Germany). Supernatants were screened for eosinophil binding by Western blot analysis (group 1, 2), flow cytometry (group 3 mice) and immunocytochemical staining of MAL cytospots (groups 1 and 3). Hybridomas showing reactivity with eosinophils but not other leukocytes were cloned by limiting dilution at least three times. The isotype of the resultant mAbs was determined using a mouse mAb isotyping kit (Isostrip, Roche Diagnostics, New South Wales, Australia).

### SDS-PAGE and Western blotting

Hybridoma supernatants obtained following the fusion of spleens from mice immunised with an eosinophil soluble or granule extract were screened by Western blot analysis. Eosinophil and neutrophil extracts were heat denatured in reducing sample buffer and subjected to SDS-PAGE on 12.5% gels without a gel comb. Cell preparations of eosinophils, lymphocytes, neutrophils and macrophages were separated by 12.5% SDS-PAGE and transferred to 0.45 μM nitrocellulose membranes (MSI, Victoria, Australia) by electroblotting at 100 V for 1 h. For screening hybridoma supernatants, the membrane was cut into strips prior to probing. Membranes were incubated with mAb supernatant for 1 h, followed by incubation with horseradish peroxidase (HRP)-conjugated rabbit anti-mouse Ig (DAKO, California, USA) for 1 h. Bound conjugate was visualised using 1.5 mM 3,3'-diaminobenzidine tetrahydrochloride (DAB; Sigma). Molecular weights were determined using SeeBlue Plus 2 prestained molecular weight markers (Invitrogen, Life Technologies, Victoria, Australia).

### Flow cytometry

Flow cytometry was used to obtain a 100% pure eosinophil preparation from MAL cells by the gating of eosinophils based on their forward scatter (FSC) and side scatter (SSC) properties. Hybridoma supernatants were screened approximately two weeks following fusion for the presence of antibodies by flow cytometry. Flow cytometry was also used for the detection of cell surface and intracellular molecules on MAL cells and PBLs as previously described [10,17]. Briefly, cells were incubated with undiluted mAb supernatants for 30 min at 4°C then centrifuged, washed and incubated with fluorescein isothyocyanate (FITC)-conjugated anti-mouse F(ab')2 immunoglobulin (Silenus, Melbourne, Australia) for 30 min at 4°C. Cells were then washed and fixed in 3% formaldehyde in PBS prior to analysis by flow cytometry. Different leukocyte cell populations were gated out on the basis of FSC and SSC characteristics and 10,000 events were analysed using a FACSCaliber™ flow cytometer (Becton-Dickinson, Mountain View, California, USA) and CellQuest™ software (Becton-Dickinson). Percentage reactivity (positive staining) and relative mean fluorescence intensity (RMFI) was assessed relative to an isotype-matched negative control mAb (SBU-3) that does not react with sheep leukocytes [10].

### Immunocytochemistry

For cytospots preparations, blood and MAL cells were fixed in 95% ethanol for 10 min. Slides were blocked for endogenous peroxidase by immersion in PBS/0.03% H_2_O_2 _for 10 min then covered with undiluted mAb supernatant and incubated in a humid box for 1 h at room temperature (RT). Slides were washed in PBS, then incubated with HRP-conjugated rabbit anti-mouse IgG (DAKO) in PBS containing 2% normal sheep serum (NSS) for 1 h. Conjugate antibody was detected by incubating slides with DAB solution for 10 min. The slides were then counterstained with haematoxylin and eosin Y (H&E; Sigma).

For immunostaining of tissue sections, tissues were either fixed in 95% cold ethanol then processed to paraffin, or embedded in Optimal Cutting Temperature (OCT) solution (Tissue Tek, Miles Inc., USA), snap-frozen in liquid nitrogen and stored at -70°C. Frozen tissue sections (5 μM) were cut onto glass microscope slides, air-dried and fixed in 95% ethanol for 10 min. Sections (paraffin and frozen) were blocked for endogenous peroxidase and non-specific binding sites were blocked by incubation with 10% NSS in PBS for 20 min at RT. Undiluted mAb supernatant was then applied and sections incubated for 60 min at RT, and, after washing in PBS, slides were incubated with biotinylated rabbit anti-mouse Ig (DAKO) for 30 min at RT. Sections were again washed, followed by a 30 min incubation in streptavidin-HRP (Silenus). Sections were developed with DAB and counterstained with H&E.

## Competing interests

The author(s) declares that there are no competing interests.

## Authors' contributions

GMS collected and analysed the data and wrote the paper.

ARY oversaw the collection and analysis of data and revised the manuscript.

ENTM originated the idea for the research and revised the manuscript.

RJB supervised the collection and analysis of the data and participated in writing the manuscript.

All authors read and approved the final manuscript.
